# Cross-reactivity of anti-HMGB1 antibodies for HMGB2

**DOI:** 10.1016/j.jim.2018.02.006

**Published:** 2018-05

**Authors:** Jessica E. Davies, Bonita H.R. Apta, Matthew T. Harper

**Affiliations:** Department of Pharmacology, University of Cambridge, Cambridge, UK

## Abstract

HMGB1 and HMGB2 are DNA-interacting proteins but can also have extracellular actions during inflammation. Despite their relatively high homology, they may have distinct roles, making it essential to be able to differentiate between the two. Here we examine the specificity of five commercially-available anti-HMGB1 antibodies. By Western blotting of recombinant proteins and HMGB1−/− mouse embryonic fibroblasts, we identified only one HMGB1 antibody that, under our experimental conditions, did not also detect HMGB2. Selecting specific antibodies for HMGB1 and HMGB2 allowed identification of distinct HMGB1 and HMGB2 subcellular pools in primary neutrophils.

## 1 Introduction

HMGB1 and HMGB2 are members of the high mobility group box (HMGB) protein family. Typically, these proteins aid DNA replication, repair and transcription. However, HMGB1 shuttles between the nucleus and cytoplasm. Cytosolic HMGB1 can be released from the cell through active secretion, or passively released when membrane integrity is compromised (Scaffidi et al., 2002). Extracellular HMGB1 has been extensively documented to act as an inflammatory cytokine (Yamada and Maruyama, 2006). Although extracellular HMGB2 is relatively understudied, it may also have extracellular actions (Pusterla et al., 2009).

Despite the high degree of sequence homology, and their structural and biochemical similarities, HMGB1 and HMGB2 functions are not identical. In knock-out studies, for example, HMGB1−/− but not HMGB2−/− mice die shortly after birth due to hypoglycaemia (Calogero et al., 1999), while HMGB2−/− males have reduced fertility (Ronfani et al., 2001). In humans, an increased serum level of HMGB1 has been implicated in several inflammatory and autoimmune diseases (Dupire et al., 2012), whereas overexpression of HMGB2 is associated with tumour aggression in certain kidney and breast carcinomas (Kwon et al., 2010). In summary, there are now many recent studies identifying novel, non-redundant roles for HMGB1 and HMGB2.

A range of monoclonal and polyclonal antibodies have been generated for HMGB1 and HMGB2 and are commercially available. These antibodies are often marketed as specific for HMGB1 or 2. In this study, we screened five commercially-available HMGB1 antibodies for specificity, evaluated through recombinant proteins and knock-out mouse embryonic fibroblasts. We found that most HMGB1 antibodies also readily detected recombinant HMGB2 to some extent under our experimental conditions. Having identified specific antibodies for HMGB1 and −2, we found that these proteins have unique cellular locations in primary human neutrophils but not in human endothelial cells, highlighting the need to be able to distinguish between these isoforms.

## 2 Materials and methods

### 2.1 Tissue culture

Human umbilical cord vein endothelial cells (HUVEC; PromoCell; c-12203) were cultured in endothelial cell growth medium (PromoCell, c-22010), containing 35 μg/ml gentamycin, 2% v/v fetal calf serum (FCS) and endothelial cell growth supplements (Promocell, c-39215). WT and HMGB1−/− mouse embryonic fibroblasts (MEF) were kindly provided by Professor S. Lippard (Massachusetts Institute of Technology, Cambridge, MA). HMGB2−/− MEFs were purchased from HMGBiotech (HM-251). MEFs were cultured in DMEM high glucose (Sigma) with 1% v/v penicillin/streptomycin (Sigma) and 5% v/v FCS.

### 2.2 Western blotting

**Fig. 1:**
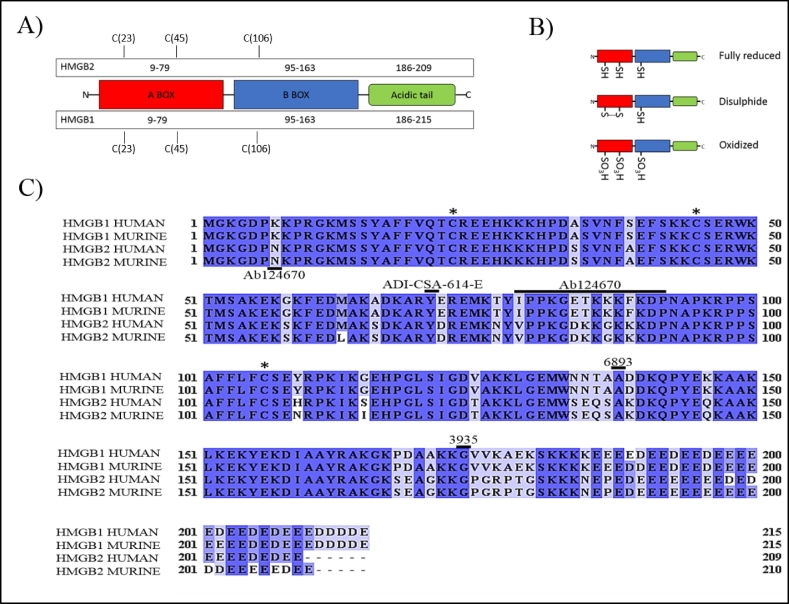
Antibody epitope identification. A) Schematic highlighting HMGB protein domains and conserved cysteine residues. B) Schematic demonstrating the three redox states of HMGB proteins. C) Human and murine HMGB1 and HMGB2 amino acid alignments, shading is co-ordinated to represent degree of similarity, while unshaded regions indicate a residue difference. Asterisk (*) defines conserved cysteine residues. Underlined residues locate the reported epitope regions (or central amino acid) for antibodies used within the screen. Note: catalogue number not clone number used as the antibody reference.

Lysates were made in RIPA lysis buffer and a protein inhibitor cocktail (Sigma) was added to prevent protein degradation. Prior to gel loading, lysate concentrations were established via Bradford assay. MEF lysates, HUVEC, neutrophil lysates (10 μg) or 1 μg recombinant proteins (ProSpec-PRO-581 & HMGBiotech-HM-153) were separated by SDS-PAGE on 12% acrylamide gels under reducing or non-reducing conditions, as indicated in the figure legend. Proteins were transferred to a PVDF membrane (MerckMillipore). Membranes were blocked for 1 h at room temperature with 5% w/v BSA (Fraction V; Sigma) in TBST (Tris-buffered saline containing 0.1% Tween-20), after which membranes were incubated with a range of HMGB1 antibodies (see Fig. 1 and S.1) or anti-HMGB2 antibodies (Rabbit monoclonal EPR6301; Abcam Cat# ab124670; RRID: AB_10975355) at 4 °C for overnight incubation. After washing with TBST and a 1 h 5% w/v milk block, membranes were incubated for 2 h at room temperature with anti-rabbit HRP (Cell Signaling Technology Cat# 7074) 1:5000, or anti-mouse HRP (Cell Signaling Technology Cat# 7076) 1: 10,000, as appropriate. When appropriate, anti-beta actin (Clone 8H10D10; Cell Signaling Technology Cat# 3700, RRID:AB_2242334) or Anti-GAPDH (Clone 14C10; Cell Signaling Technology Cat# 5014, RRID:AB_10693448) were used as loading controls. Images were collected through chemiluminescent substrate chemistry (Thermofisher Cat# 34577) and documented on X-ray film (Amersham Hyperfilm ECL, VWR, Cat# 28–9068-35) and developed using an OptiMax X-ray film processor (Protec Medizintechnik).

### 2.3 Primary cell isolation

Neutrophils were obtained from healthy volunteers who provided their informed, written consent (local ethics committee approved). Blood was collected in 4% sodium citrate. To isolate neutrophils, 25 ml of blood was layered on 15 ml Histopaque-1077. Following centrifugation (400 ×*g*, 30 min, no break), the buffy coat layer was removed to leave the erythrocyte/neutrophil pellet. The pellet was resuspended 1:1 v/v in Hanks buffered saline solution (HBSS, without Ca^2+^ and Mg^2+^; Sigma), then diluted 1:1 in 2% Dextran-500 (made up in HBSS). Erythrocytes were then left to settle for 20 min at room temperate before the upper (neutrophil layer) was collected, diluted and pelleted in HBSS. The cell pellet was resuspended in ddH_2_O for 30 s to lyse residual erythrocytes, after which the solution was diluted in 50 ml HBSS and pelleted for immediate resuspension. Neutrophils were used within 4 h of isolation.

### 2.4 Immunofluorescence

HUVEC and MEF were grown to confluence on glass coverslips prior to 4% paraformaldehyde (PFA) fixation for 20 min at room temperature. After washing with PBS, cells were permeabilised with 0.1% Triton-X100 for 10 min. Neutrophils were fixed (1% PFA) in suspension to avoid activation with the glass coverslip, and permeabilised as above. Neutrophils were then left to adhere for a minimum of 2 h at 4 °C to poly-l-lysine-treated glass coverslips. After PBS washing, all samples were incubated with anti-HMGB1 (CST 3935, 1:100) or anti-HMGB2 (Ab124670, 1:100) primary antibodies and left overnight in 33% v/v FCS and 0.1% v/v sodium azide. Primary antibodies were removed, and fluorophore-conjugated anti-rabbit antibodies (Invitrogen Cat# 11-4839-81) 1:200 were applied for 1 h at room temperature. Fluorescent images were acquired by confocal scanning laser microscopy (Leica). Microscope settings were optimised and kept constant for each cell type.

## 3 Results

### 3.1 Epitope identification of HMGB1 antibodies

Human amino acid sequences of HMGB1 and HMGB2 were sourced from the National Centre for Biotechnology Information (NCBI). Amino acid sequences were aligned using the free software, Jalview (Clamp et al., 2004) to identify regions of homology (Fig. 1C). Using Jalview, it was calculated that there is 79% sequence similarity between human HMGB1 and human HMGB2. Both proteins contain two homologous lysine-rich DNA-binding domains, A-Box and B-Box, and a highly negatively-charged C-terminal tail (Fig. 1A). Within the HMGB protein there are three conserved cysteine residues (Fig. 1A). These play a key role in producing unique redox forms, (Fig.1B), which regulate the biological effects of release HMGB proteins (Erlandsson Harris et al., 2012).

There were three criteria for choosing antibodies for the screen: 1) the antibody should be commercially and readily available from a mainstream scientific retailer; 2) the antibody should be classified as specific (in particular, there should be no apparent cross-reactivity with HMGB2); 3) there should be minimal overlap wherever possible with epitopes between antibodies used in the screen. Epitope regions for HMGB1 were identified for each HMGB1 antibody within the screen by contacting the manufacturers (Table 1). Unfortunately, due to protection of intellectual property many of the immunogen sequences were not divulged from the manufacturers. In these cases, centralised amino acids of the epitopes were given. In the case of the antibody MAB1690, R&D Systems stated that they do not conduct epitope mapping so were unable to locate the exact address of their antibody.

**Table 1:** Antibody characteristics. CST, Cell Signaling Technology.

Catalogue number	Clone	Company	RRID	Species	Monoclonal/Polyclonal	Epitope reported by vendor
Ab92310	EPR3506	Abcam	AB_10975355	Rabbit	Monoclonal	IPPKGETKKKFKDP
6893	D3E5	CST	AB_10827882	Rabbit	Monoclonal	Alanine 137
3935	N/A	CST	AB_2295241	Rabbit	Polyclonal	Glycine 174
ADI-CSA-614-E	KS1	Enzo Life Sciences	AB_10631414	Mouse	Monoclonal	Arginine 70-Glutamine 72
MAB1690	115603	R&D Systems	AB_2117897	Mouse	Monoclonal	Not available
Ab124670	EPR6301	Abcam	AB_10975355	Rabbit	Monoclonal	Asparagine 7

### 3.2 HMGB1-specific antibody selection

**Fig. 2:**
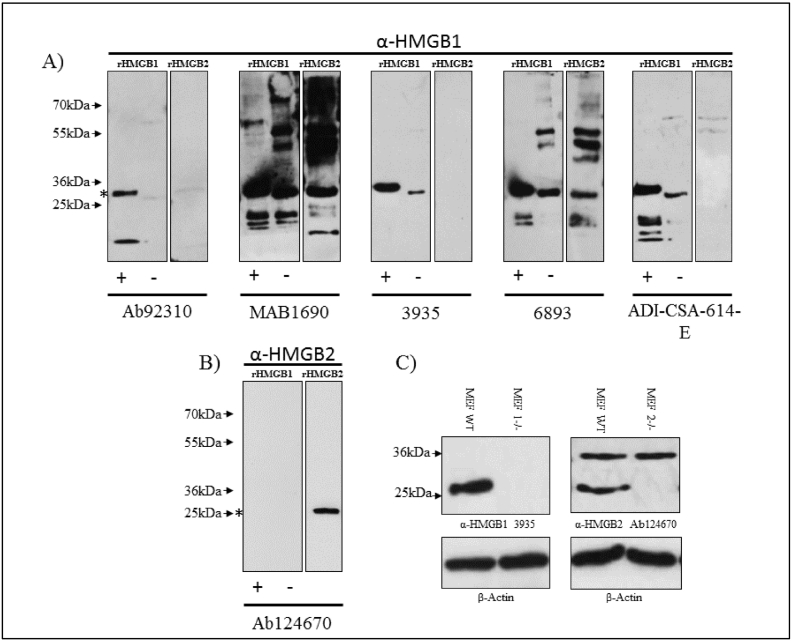
HMGB1 and HMGB2-specific antibody selection. A + B) Western blot of human recombinant (r)HMGB1 and rHMGB2. rHMGB1 was probed under reducing (+) and non-reducing (−) conditions. All membranes were exposed for the same time to ensure comparability. Asterisks (*) on the left-hand side indicate the expected weight of his-tagged rHMGB1 and non-tagged rHMGB2. C) Western blot of wildtype (WT), HMGB1−/− (MEF 1−/−) and HMGB2−/− (MEF2−/−) MEF lysates, under reducing conditions. Images are representative of 3 independent repeats.

His-tagged, recombinant human HMGB1 (~28 kDa) and (untagged) recombinant human HMGB2 (~25 kDa) were subjected to SDS-PAGE and Western blotting with each antibody. All antibodies were used at their recommended concentration (Fig. S.1). Under our experimental conditions, MAB1690 and 6893 readily detected HMGB2 in addition to HMGB1 (Fig. 2A). Ab92310 weakly detected HMGB2. More intense bands were observed under reducing conditions against the recombinant HMGB1, suggesting that all antibodies in this screen displayed higher affinity for the reduced redox form of HMGB1. The non-reduced protein ran slightly faster, suggesting that the single disulphide bond influences the globular structure of the protein. Notably, Ab92310 could readily detect reduced HMGB1 but was particularly poor at detecting non-reduced HMGB1. This means that Ab92310 might not detect native HMGB1 if present in its non-reduced form. Some HMGB1 antibodies (MAB1690 and 6893) detected prominent bands at around 55 kDa and 50 kDa. This may represent dimers or aggregates forming at high concentrations of recombinant HMGB1 protein. These bands were also weakly detected by ADI-CSA-614-E, which also detected similar bands with recombinant HMGB2 (despite not detecting monomer HMGB2). Low molecular weight fragments were observed with all HMGB1 antibodies except for 3935. This may represent recombinant protein degradation products. In most cases these were only observed in reduced samples. Based on these observations, we decided to use 3935 as our HMGB1-specific antibody.

Additionally, one HMGB2 antibody was trialled (Ab124670).This antibody did not detect HMGB1 under our experimental conditions, either in reduced or non-reduced forms, but readily detected HMGB2, suggesting that it does specifically detect HMGB2. Interestingly, Ab124670 is reported to bind to an epitope that varies by only one amino acid between HMGB1 and HMGB2 in both mice and humans (Fig. 1).

To test whether these antibodies retained specificity when used on cell lysates, we took advantage of previously generated knock out MEF HMGB1−/− and HMGB2−/− cell lines (Fig. 2B). Given the high degree of sequence likeness for human and murine HMGB1 and HMGB2, 99% and 97% respectively (Fig. S.2), it was assumed that MEF cells would be a reliable cell line to work with. Anti-HMGB1 3935 readily detected HMGB1 in wild-type (WT) MEFs. This band was absent in HMGB1−/− MEFs, and no other bands were observed. Conversely, anti-HMGB2 Ab124670 readily detected a ~25 kDa protein in WT MEFs that was absent in HMGB2−/− MEFs. In addition, there was a non-specific band at ~36 kDa when MEFs were probed with Ab124670.

### 3.3 HMGB1 is differentially located in neutrophils compared to HMGB2

**Fig. 3:**
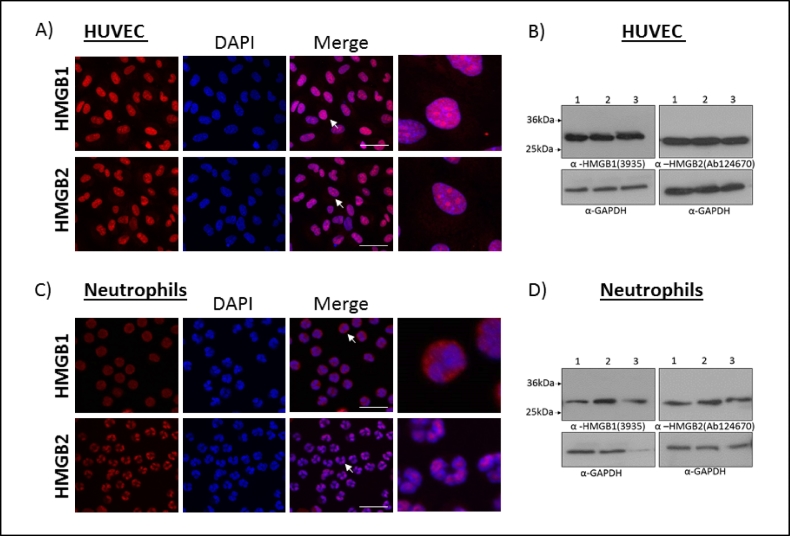
Subcellular locations of HMGB1 and HMGB2 are cell type specific. Confocal microscopy images of HUVEC (A) and neutrophils (B) using a 63× oil immersion lens. HMGB1 (3935) and HMGB2 (EPR6301) proteins are shown in red, and blue is the nuclear stain (DAPI). Scale bar is 50 μm in both. The cell marked with the white arrow in the merge is shown in higher magnification in the final panel. Images are representative of 3 independent repeats. Western blot of HUVEC (C) and neutrophil (D) reduced lysates. 1–3 indicate three different lysates prepared independently. (For interpretation of the references to colour in this figure legend, the reader is referred to the web version of this article.)

To demonstrate the importance of specific antibodies for HMGB1 and HMGB2, endothelial cells (HUVEC) and primary human neutrophils were stained to identify HMGB1 and HMGB2 cellular locations by confocal microscopy. HMGB1 and HMGB2 were both nuclear in HUVEC (Fig. 3A). In contrast, HMGB1 and HMGB2 had distinct locations in neutrophils. HMGB1 in resting neutrophils was predominantly cytosolic and distributed in puncta, whereas HMGB2 was retained in the nucleus (Fig. 3B). Appropriate IgG controls were carried out (Fig. S.3). Finally, we tested whether the two antibodies used could specifically detected HMGB1 and HMGB2 in HUVEC and neutrophils by Western blot (Fig. 3C–D). A single band (~25 kDa) was observed for each protein in each cell type. Notably, the non-specific band (~36 kDa) found when MEF lysates were probed with anti-HMGB2 (Ab124670) was not seen in either HUVEC or neutrophil lysates. Cytoplasmic HMGB1 in neutrophils has been previously shown to be due to mono-methylation of Lys42 (Ito et al., 2007). However, to the best of our knowledge this is the first example of HMGB1 and HMGB2 being differentially localised within the same cell, which leads to speculation that these 79% homologous proteins may have different biological functions in neutrophils.

In conclusion, some commercially-available HMGB1 antibodies cross-reacted with the highly homologous recombinant protein, HMGB2, under our experimental conditions. We selected two specific HMGB1 and HMGB2 antibodies based on Western blot analysis from recombinant proteins and MEF lysates deficient in HMGB1 or HMGB2. By using these specific antibodies, we demonstrated that although HMGB1 and HMGB2 are identically localised to the nucleus in endothelial cells, in neutrophils HMGB2 remained nuclear and HMGB1 cytoplasmic. This suggests that HMGB1 and 2 may have distinct roles in neutrophils, and highlights the importance of antibody specificity, especially when working with primary cells which are not genetically tractable.

## Appendix A Supplementary data

Supplementary material Image 1
