# Supplementary material for: Cross-reactivity of anti-HMGB1 antibodies for HMGB2
Source: J Immunol Methods. 2018 May;456:72–6. doi: 10.1016/j.jim.2018.02.006 (PMC5886380; doi:10.1016/j.jim.2018.02.006)
Supplement: Supplementary file 1 — Supplementary material [file mmc1.docx]

Supplemental data:


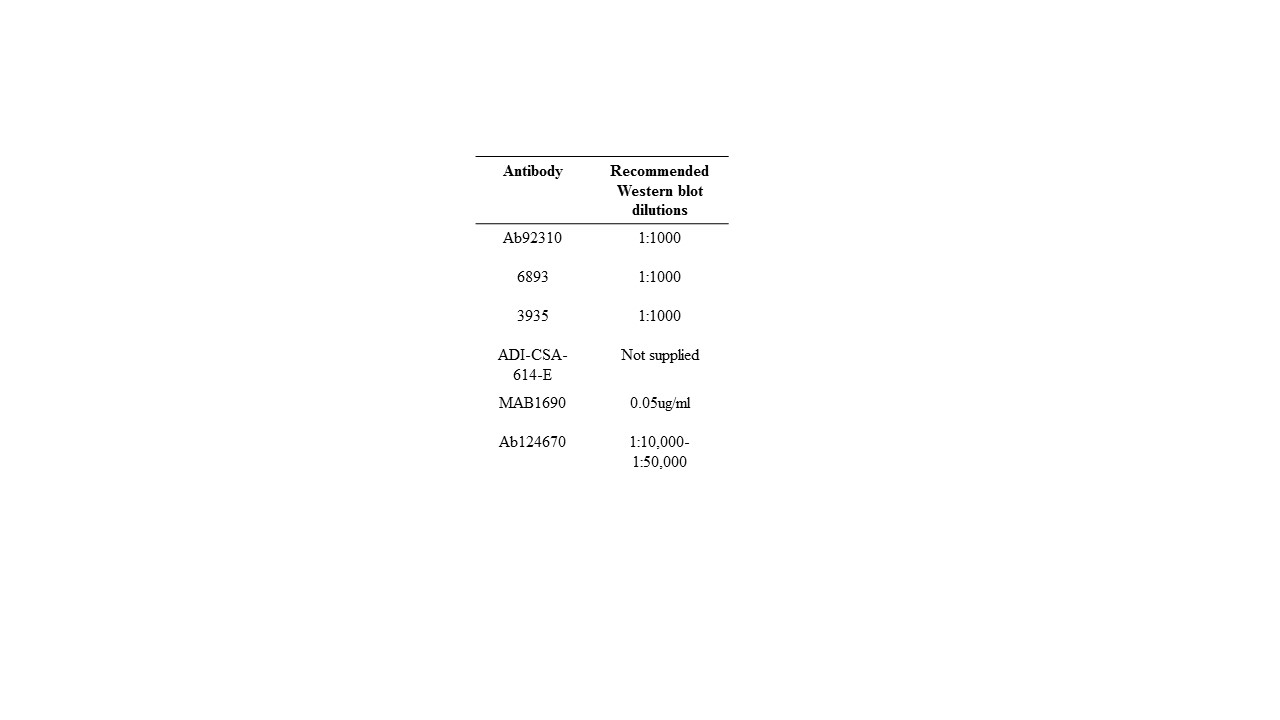


S.1 Table of recommended antibody dilutions. Information taken from antibody datasheets. As there was no recommended dilution for ADI-CSA-614-E it was used at 1:1000 dilution.


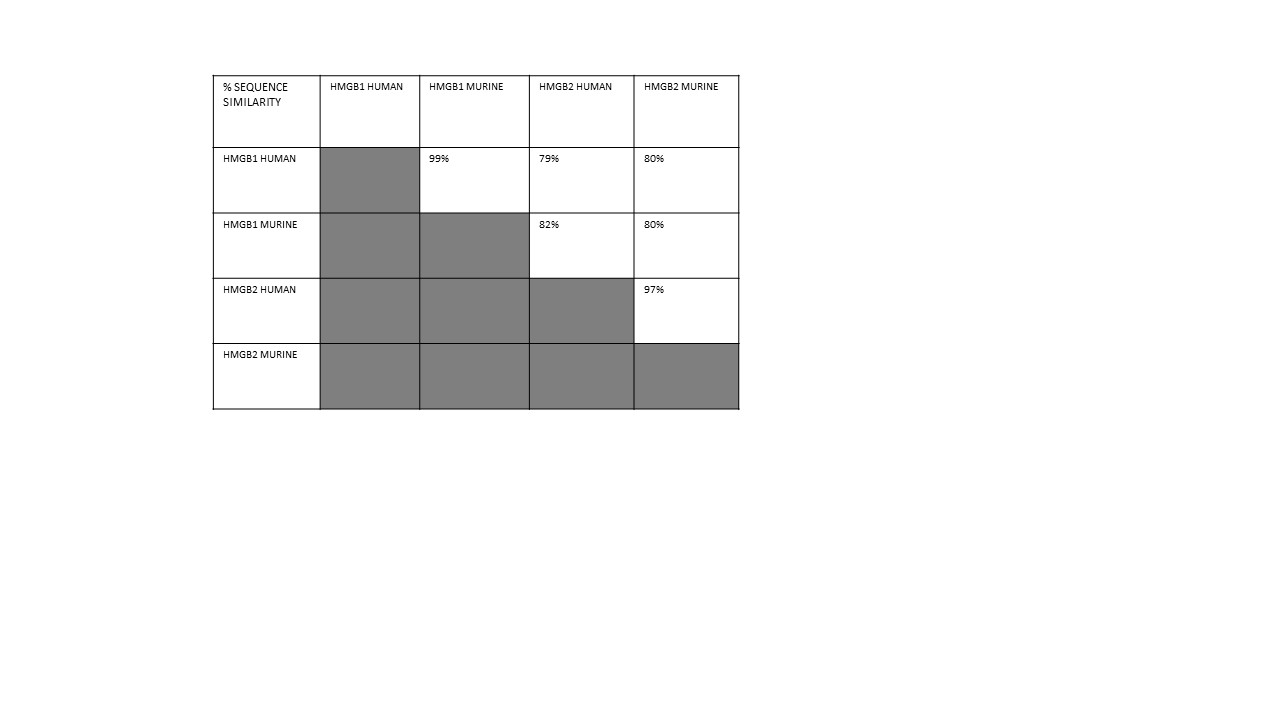


S.2 Table of amino acid sequence similarity. Data describing percentage sequence similarity between homologues and orthologues. Alignments were conducted using Jalview.


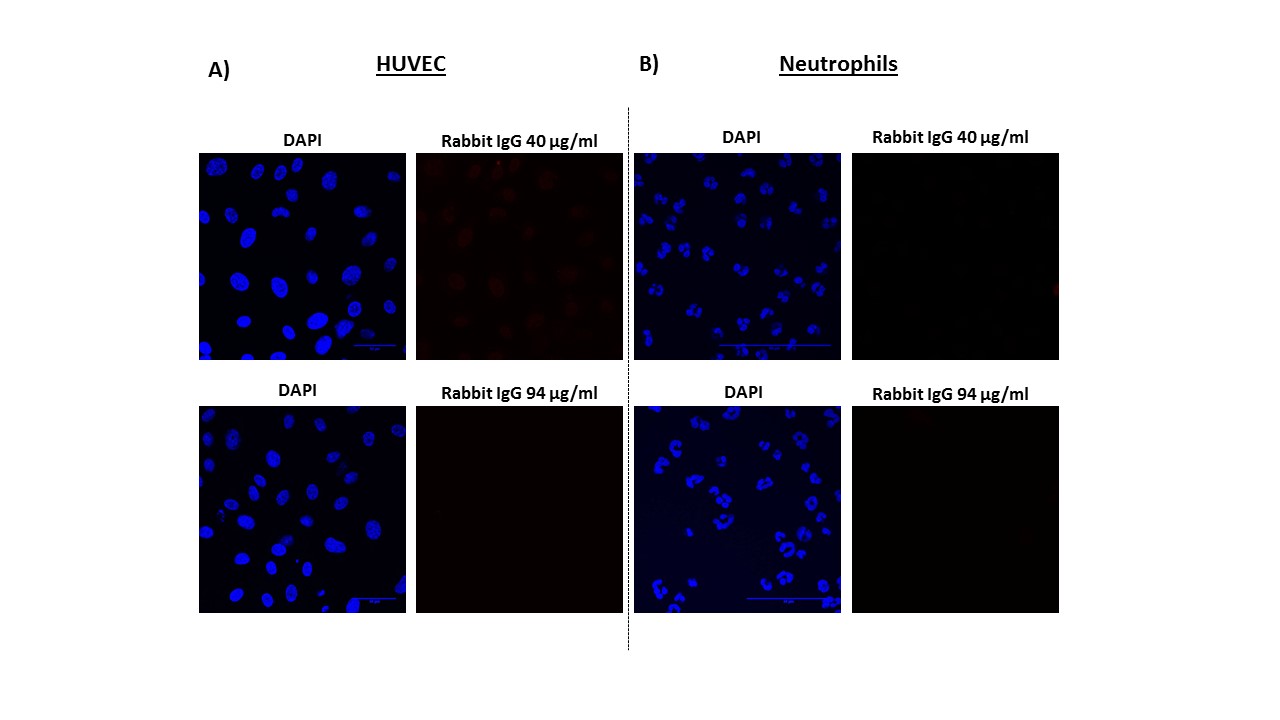


S.3. IgG control confocal microscopy experiments. Confocal microscopy images of HUVEC (A) and neutrophils (B) using a 63x oil immersion lens. Cell were fixed and permeabilised as described in section 2.4, and incubated with rabbit IgG control (CST 27295) at either 40μg/ml or 94μg/ml to mimic anti-HMGB1(3935) or anti-HMGB2(Ab124670) concentrations respectively. Post primary antibody incubation cells were washed and stained with 1:200 anti-rabbit (Invitrogen 11-4839-81) for 1 hour at room temperature. An extremely low IgG signal indicates that there is minimal none specific antibody binding in this assay.
